# Effectiveness of the 10-valent pneumococcal conjugate vaccine against radiographic pneumonia among children in rural Bangladesh: A case-control study

**DOI:** 10.1016/j.vaccine.2020.08.035

**Published:** 2020-09-29

**Authors:** Eric D. McCollum, Salahuddin Ahmed, Arun D. Roy, Nabidul H. Chowdhury, Holly B. Schuh, Syed J.R. Rizvi, Abu A.M. Hanif, Ahad M. Khan, Arif Mahmud, Farhan Pervaiz, Meagan Harrison, Megan E. Reller, Nicole Simmons, Abdul Quaiyum, Nazma Begum, Mathuram Santosham, William Checkley, Lawrence H. Moulton, Abdullah H. Baqui, For the Projahnmo Study Group in Bangladesh

**Affiliations:** a Global Program in Respiratory Sciences, Eudowood Division of Pediatric Respiratory Sciences, Department of Pediatrics, School of Medicine, Johns Hopkins University, Baltimore, USA; b Health Systems Program, Department of International Health, Bloomberg School of Public Health, Johns Hopkins University, Baltimore, MD, USA; c Johns Hopkins University – Bangladesh, Dhaka, Bangladesh; d Global Disease Epidemiology and Control Program, Department of International Health, Bloomberg School of Public Health, Johns Hopkins University, Baltimore, MD, USA; e Division of Pulmonary and Critical Care, Department of Medicine, School of Medicine, Johns Hopkins University, Baltimore, MD, USA; f Center for Global Non-Communicable Disease Research and Training, School of Medicine, Johns Hopkins University, Baltimore, MD, USA; g Division of Infectious Diseases, Department of Medicine, Duke University School of Medicine, Durham, NC, USA; h Duke Hubert-Yeargan Center for Global Health, Durham, NC, USA; i Duke Global Health Institute, Durham, NC, USA; j Maternal and Child Health Division, International Centre for Diarrhoeal Disease Research, Bangladesh (icddr,b), Dhaka, Bangladesh

**Keywords:** Pneumococcal vaccines, Respiratory tract infections, Radiography, Asia, Bangladesh, Child, Infant

## Abstract

**Background:**

Pneumococcal conjugate vaccine (PCV) effectiveness against radiographic pneumonia in South Asia is unknown. Bangladesh introduced PCV10 in 2015 using a three dose primary series (3 + 0). We sought to measure PCV10 effectiveness for two or more vaccine doses on radiographic pneumonia among vaccine-eligible children in rural Bangladesh.

**Methods:**

We conducted a matched case-control study over two years from 2015 to 2017 using clinic and community controls in three subdistricts of Sylhet, Bangladesh. Cases were vaccine eligible 3–35 month olds at Upazila Health Complex outpatient clinics with World Health Organization-defined radiographic primary endpoint pneumonia (radiographic pneumonia). Clinic controls were matched to cases within a one week time window by age, sex, and clinic and had an illness unlikely to be Streptococcus pneumoniae; community controls were healthy and similarly matched within a one week time window by age and sex, and distance from the clinic. We estimated adjusted vaccine effectiveness (aVE) using conditional logistic regression.

**Results:**

We matched 1262 cases with 2707 clinic and 2461 community controls. Overall, aVE using clinic controls was 21.4% (95% confidence interval, −0.2%, 38.4%) for ≥2 PCV10 doses and among 3–11 month olds was 47.3% (10.5%, 69.0%) for three doses. aVE increased with higher numbers of doses in clinic control sets (p = 0.007). In contrast, aVE using community controls was 7.6% (95% confidence interval, −22.2%, 30.0%) for ≥2 doses. We found vaccine introduction in the study area faster and less variable than expected with 75% coverage on average, which reduced power. Information bias may also have affected community controls.

**Conclusions:**

Clinic control analyses show PCV10 prevented radiographic pneumonia in Bangladesh, especially among younger children receiving three doses. While both analyses were underpowered, community control enrollment – compared to clinic controls – was more difficult in a complex, pluralistic healthcare system. Future studies in comparable settings may consider alternative study designs.

## 1 Introduction

Pneumococcus, or *Streptococcus pneumoniae,* is an important etiology of childhood pneumonia and is a leading cause of death globally among children less than five years old [1], [2], [3]. According to the most recent 2015 estimates, 48% of the 8.9 million pediatric pneumococcal pneumonia cases that occur each year globally occur in the Southeast Asian region [4]. Bangladesh, a densely populated country in this region, has the tenth highest pneumococcal pneumonia mortality burden in under-five year old children worldwide and nearly 40% of deaths from lower respiratory tract infection in under-five year old children in Bangladesh are attributed to *S. pneumoniae* [2].

As of 2018, 142 countries had introduced pneumococcal conjugate vaccine (PCV) into their national immunization schedules [5]. Seminal PCV trials from low-income and middle-income countries (LMICs) including The Gambia [6], South Africa [7], and Philippines [8] demonstrated a vaccine efficacy against radiographic-confirmed pneumonia, defined according to World Health Organization (WHO) criteria as primary endpoint pneumonia on chest radiography [9], among under-five year old children with an acute respiratory illness of 37%, 25%, and 23%, respectively. With assistance from Gavi and the Vaccine Alliance, 10-valent PCV (Synflorix™, GlaxoSmithKline) was introduced into Bangladesh in March 2015 [10], [11]. The effectiveness of PCV introduction against WHO-defined radiographic primary endpoint pneumonia among children in Bangladesh was unknown.

The WHO recommends PCV impact evaluations during vaccine introduction into national programs as well as ongoing pneumococcal disease surveillance post PCV introduction [12]. These data are essential for informing future investment into national PCV immunization programming, especially for countries that have already or will soon graduate from Gavi and therefore use their own financial resources to immunize children, for monitoring for pneumococcal serotype replacement, and for estimating the potential impact of PCV introduction into neighboring countries with similar disease epidemiology. Given most LMICs lack resources to indefinitely sustain surveillance systems for monitoring radiographic pneumonia incidence, case-control study designs have been recommended as an alternate approach [13] and have been applied successfully in sub-Saharan Africa [14], [15]. To evaluate PCV10 effectiveness against radiographic pneumonia among vaccine-eligible children in Bangladesh, we conducted a large-scale population-based case-control study across a northeast district from 2015 to 2017. Our primary objective was to measure the effectiveness of two or more doses of PCV10 on radiographic pneumonia among 3–35 month old vaccine-eligible children using clinic and community control analyses.

## 2 Methods

### 2.1 Study design

This is a matched case-control study of children with first-episode WHO radiographic pneumonia with clinic controls and community controls.

### 2.2 Study setting

The Projahnmo research group conducted the study between October 1, 2015 and September 30, 2017 in three subdistricts (upazilas) of Syhlet district, northeast Bangladesh. Projhanmo is a research partnership of Johns Hopkins University with the Government of Bangladesh’s Ministry of Health and Family Welfare (MOHFW) and Bangladeshi non-governmental and academic institutions.

**Fig. 1:**
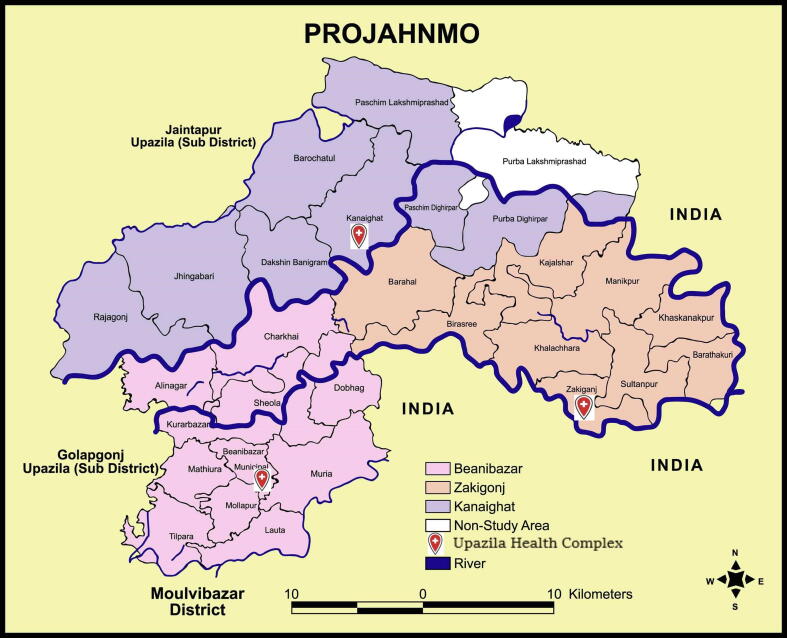
PROJAHNMO study area.

The study area and population surveillance methods have been previously described in detail [10]. In brief, the Zakiganj, Kanaighat, and Beanibazar upazilas included in the study have an overall population of about 770,000 and annual birth cohort of approximately 20,000 (Fig. 1). Surveillance infrastructure included a team of trained female community health workers (CHWs), an updated census, and real-time Global Positioning System mapping. CHWs are community residents who visit all households in an area of about 10,000 individuals every two months to promote illness recognition and care seeking, to update population data, to monitor women for pregnancy, and to assess children for respiratory illnesses. For this study, surveillance was intensified to weekly visits in a random 13% subset of households to improve timely detection of respiratory illness. If a child was found to have an acute illness, then the CHW referred the child to the nearest MOHFW clinic. One MOHFW under-five outpatient clinic at each subdistrict’s Upazila Health Complex participated. We staffed under-five clinics of each Upazila Health Complex with study physicians and started computed radiology (CR) in in 3–35 month old children with a possible lower respiratory infection using an analogue unit (POLYMOBIL® Plus, Siemens, Erlangen, Germany) and CR Fuji Film cassette reader to digitize images. In March 2015 the national immunization program introduced a schedule of PCV10 to be given to children at six, 10, and 18 weeks of age with a one-year catch-up campaign for infants <12 months in the study area. In April 2017 the schedule was changed to six, 10, and 14 weeks.

## 3 Participants

### 3.1 Cases

Cases were vaccine age-eligible children 3–35 months old from the surveillance area with WHO-defined radiographic primary endpoint pneumonia (radiographic pneumonia) at one of the three Upazila Health Complexes and were residents in the surveillance area. Study physicians at the clinics were trained to identify children with possible lower respiratory infection as potential cases according to the study protocol. Possible lower respiratory infection was defined as cough or difficult breathing (by history or observation) and either fast breathing (respiratory rate ≥50 breaths/minute for 3–11 month olds and ≥40 breaths/minute for 12–35 month olds) or a clinical sign of respiratory illness (including lower chest wall indrawing, persistent nasal flaring, cyanosis, head nodding or tracheal tugging, grunting, stridor while calm, or crackles or wheeze on chest auscultation). After written informed consent children meeting these criteria had a supine antero-posterior chest radiograph obtained at the Upazila Health Complex. Study physician performance was monitored monthly by the study’s pediatric pulmonologist (EDM). Refresher trainings were provided every six months by EDM, and remediation was conducted as needed.

A chest radiograph reading panel of eight Bangladeshi radiologists and pediatricians was formed to interpret images according to WHO chest radiograph methods for vaccine studies [9]. Each reader was trained and calibrated to the WHO chest radiograph definitions at the study start by a WHO-certified trainer and every six months by a pediatric pulmonologist (EDM); both are members of the WHO Chest Radiography in Epidemiological Studies technical working group [9]. Readers not meeting WHO calibration standards or study quality control measures were remediated. Readers were blinded to patient clinical data and image interpretations from other readers. Each chest radiograph was allocated randomly to two readers. If the readers agreed the image was interpretable and did or did not fit the WHO definition of radiographic primary endpoint pneumonia, then the classification was final. If the interpretations by the two readers were discordant then another reader was randomly allocated to review the image as an arbitrator. If the arbitrator’s interpretation was consistent with one of the initial readers then the classification was considered final, however, if the interpretation was also discordant with the other readers then a second arbitrator (EDM) finalized the classification. Only one reader was required to detect WHO-defined “other infiltrate.”

After the chest radiograph reading panel identified a child as a case, the image results were shared with the MOHFW study physicians and parents. Study data collectors visited case households within one month of selection to collect additional medical history, demographic and household characteristics, and PCV status. PCV status was ascertained from the child’s immunization card and a digital copy of the card was generated for quality control. In the event the immunization card was unavailable, the caregiver was asked whether or not the child had received any vaccines after birth. If the caregiver reported that the child had been vaccinated then study staff visited the associated MOHFW immunization center and extracted the vaccination data from the center’s register. Children not listed in the register were excluded from this analysis. In line with recommended case-control methodology for ascertaining vaccine status, children whose caregivers indicated that they had not received any post-birth vaccinations were considered to have zero PCV doses [16].

### 3.2 Controls

Two sets of controls were identified for each case: Upazila Health Complex outpatient clinic controls and community controls. The study began with a matching ratio of 1 case: 4 clinic controls: 4 community controls but, due to a higher than anticipated case burden, the matching ratio changed to 1: 2: 2 in January 2016. Clinic controls were randomly selected from the clinic database and community controls from the population register. Detailed randomization procedures are described elsewhere [10]. All controls were matched with cases within one week on sex and age (date of birth within one month). Clinic controls visited the same MOHFW clinic within one week of the case and had an illness unlikely to be due to *S. pneumoniae* (i.e., absence of respiratory symptoms or signs, documented fever, signs of meningitis, or acute otitis media). Community controls were additionally matched on road distance from the participant’s home village to the case MOHFW clinic. The same child was permitted to be a control for more than one case.

A data collector conducted a household visit for all possible controls, targeted to within one month of case enrollment. The data collector was not blinded to whether the household visit was for a case or a potential control. At the household visit the study data collector asked the caregiver for their consent to include the child as a control. Community controls underwent a screening examination to identify any acute signs of an illness consistent with *S. pneumoniae* as well as caregiver recall of any symptoms in the preceding one week. Children who were healthy without signs and symptoms of an illness were included as a community control. The same procedures used to ascertain the PCV status of cases were used for controls.

### 3.3 Statistical analysis

We limited cases to first episodes of radiographic pneumonia. For primary analyses we defined participants to be vaccinated if they received two or more doses of vaccine. Consistent with other PCV studies, we also did not count recent PCV doses (those received within two weeks) before case or control identification given the implausibility of a sufficiently protective immunologic response [14], [15]. Study sample size assumed a two-dose PCV coverage of 46%. Vaccine coverage estimates were derived based on two-dose Pentavalent coverage in the study area. We assumed the probability of completing two PCV doses would follow a log-normal distribution starting at 10 weeks of age, with median at 15 weeks of age and 80% coverage at 52 weeks of age, with no vaccination after 52 weeks. This resulted in an estimated average vaccine coverage level of 46% over the 24-month study period. Enrolling 1130 cases, each with two controls, would therefore have 80% power to detect 20% vaccine effectiveness at a significance level of 5%.

Conditional logistic regression (Stata version 14.2, College Station, TX) was used to assess relationships between case-control status in single and multiple variable analysis. A large set of potential adjustment variables were considered, including data on demographic and socioeconomic aspects of the cases and controls and their families and family members, family decision-making dynamics, and physical aspects of the home environment. Categorical variables were combined to have at least 50 cases (about 3%) in each category; if data on a variable was missing for >5% of the cases, a category Missing was added; else, casewise deletion was used. The goal of adjusted analyses was to control for potential confounding, as opposed to identifying predictive variables, and proceeded in two stages. First, we screened variables by requiring a p-value < 0.2 or, for dichotomous variables, an odds ratio (OR) >1.5 or <0.67. Then, we entered these variables into a backward selection procedure with p > 0.2 required to remove each from the conditional logistic model. This was done for the community controls with the “at least two PCV doses” analysis, with the same set of final adjusting variables used in all models, including those using the clinic controls, to assure comparability across models. We defined vaccine effectiveness as 100% × (1-OR). Trend tests related the number of received doses to case-control status. The only subgroups specified *a priori* were those formed by case age at diagnosis: 3–11 months, and 12–35 months.

### 3.4 Ethics

The Johns Hopkins Bloomberg School of Public Health, Johns Hopkins School of Medicine, Bangladesh Institute of Child Health, and the Ethical Review Committee of the International Centre for Diarrhoeal Diseases Research, Bangladesh, Institutional Review Boards all approved the study’s protocol.

## 4 Results

**Fig. 2:**
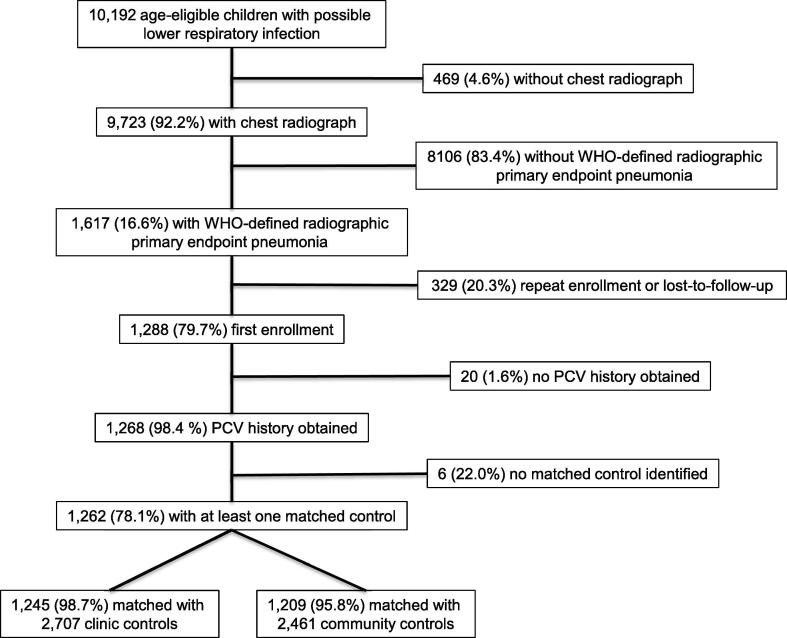
Study profile.

We found 10,192 age-eligible children that met criteria for possible lower respiratory infection (Fig. 2). Among the 9723 children that had a chest radiograph, the reading panel classified 1617 as cases with WHO-defined radiographic pneumonia. Twenty (1.6%) cases did not have a PCV history and were excluded from the analysis. Overall, 1262 cases were matched with 2707 clinic controls and 2461 community controls.

The median age of the 1262 cases with matched controls was 10 months and 574 (45.5%) were female (Table 1). The most frequently identified respiratory signs were fast breathing for age (n = 983, 77.9%), crackles on chest auscultation (n = 922, 73.1%), and lower chest wall indrawing (n = 453, 35.9%). An axillary temperature ≥101 °C was observed in 303 cases (24.0%).

**Table 1:** Characteristics of cases with World Health Organization-defined radiographic primary endpoint pneumonia.

		Total	Beanibazar clinic	Zakiganj clinic	Kanaighat clinic
		N = 1262	N = 387	N = 363	N = 512
Case characteristics	Age, months (median, IQR)	10 (6, 17)	10 (6, 18)	9 (5, 15)	10 (6, 16)
	Females, n (%)	574 (45.5%)	171 (44.2%)	153 (42.1%)	250 (48.8%)

Chest radiography	Primary endpoint pneumonia only, n (%)	285 (22.6%)	75 (19.4%)	81 (22.3%)	129 (25.2%)
	Primary endpoint pneumonia and other infiltrate, n (%)	977 (77.4%)	312 (80.6%)	282 (77.7%)	383 (74.8%)

Respiratory signs	Fast breathing for age, n (%)	983 (77.9%)	277 (71.6%)	266 (73.3%)	440 (85.9%)
	Chest indrawing, n (%)	453 (35.9%)	145 (37.5%)	155 (42.7%)	153 (29.9%)
	Head nodding or tracheal tugging, n (%)	73 (5.8%)	24 (6.2%)	21 (5.8%)	28 (5.5%)
	Stridor while calm, n (%)	0	0	0	0
	Cyanosis, n (%)	1 (0.1%)	1 (0.3%)	0	0
	Nasal flaring, n (%)	73 (5.8%)	28 (7.2%)	18 (5.0%)	27 (5.3%)
	Grunting, n (%)	8 (0.6%)	1 (0.3%)	7 (1.9%)	0
	Crackles on auscultation, n (%)	922 (73.1%)	274 (70.8%)	240 (66.1%)	408 (79.7%)
	Wheeze on auscultation, n (%)	6 (0.5%)	3 (0.8%)	3 (0.8%)	0

Non-respiratory signs	Axillary temperature ≥ 101 °C, n (%)	303 (24.0%)	95 (24.5%)	80 (22.0%)	128 (25.0%)
	Convulsions , n (%)	4 (0.3%)	1 (0.3%)	1 (0.3%)	2 (0.4%)
	Lethargy, n (%)	1 (0.1%)	0	0	1 (0.2%)
	Unable to eat or drink, n (%)	0	0	0	0
	Vomits everything, n (%)	0	0	0	0

IQR indicates interquartile range.

We found cases to have similar qualitative associations between matched clinic controls and community controls for several key demographic and risk factor variables (Table 2). Cases, compared to clinic and community controls, had a higher percentage of less educated mothers and exposure to second hand smoke or biomass fuel cooking, were on average poorer according to the principal component analysis wealth index, and lived in households with a higher average number of children <5 years old. A lower proportion of cases, compared to both control sets, had at least one employed caregiver and received at least one, at least two, or three doses of PCV. Three percent of community controls and 11% of clinic controls were selected as a control for more than one case.

**Table 2:** Characteristics of cases and controls in the Bangladesh case-control study of the effectiveness of PCV10 against World Health Organization-defined radiographic pneumonia.

Characteristics		Cases (N = 1262)	Clinic controls (N = 2707)	p value^a^	Community controls (N = 2461)	p value^b^
Demographics						
	Age in months, median (IQR)	10.3 (6.2, 16.8)	10.3 (6.2, 16.4)	0.826	10.7 (6.5, 16.8)	<0.001
	Female, n/N (%)	574 (45.5%)	1247 (46.1%)	NA	1118 (45.4%)	NA
	Maternal age in years, median (IQR)^3^	28.0 (24.5, 33.6)	27.5 (23.9, 32.7)	0.034	27.6 (24.1, 32.7)	0.044
Mother’s education^c^				<0.001		<0.001
	No education, n/N (%)	231 (18.3%)	337 (12.4%)		308 (12.5%)	
	Grade 1–5, n/N (%)	465 (36.8%)	849 (31.4%)		741 (30.1%)	
	Grade 6–10, n/N (%)	393 (31.1%)	1103 (40.7%)		996 (40.5%)	
	Grade 11+, n/N (%)	28 (2.2%)	129 (4.8%)		136 (5.5%)	
	Madrasha only, n/N (%)	145 (11.5%)	287 (10.6%)		280 (11.4%)	
Household	Number of people in household, mean (SD)	7.0 (3.4)	7.1 (3.5)	0.380	7.2 (3.5)	0.046
	Number of children < 5 years old in household, mean (SD)^c^	1.8 (0.8)	1.7 (0.9)	0.001	1.7 (0.8)	0.036
	Employed caregiver, n (%)	160 (12.7%)	393 (14.5%)	<0.001	363 (14.8%)	0.033
	Cigarette, bidi, or hookah smoker in household, n/N (%)	898 (71.2%)	1758 (64.9%)	<0.001	1587 (64.5%)	<0.001
	Biomass fuel exposure, n/N (%)	1195 (94.7%)	2401 (88.7%)	<0.001	2222 (90.3%)	<0.001
	Household PCA wealth index, mean (SD)	−0.9 (1.9)	−0.1 (2.2)	<0.001	0.1 (2.3)	<0.001
	Distance from clinic in km, mean (SD)	10.7 (7.4)	7.6 (6.0)	<0.001	10.6 (7.4)	0.431
Risk factors						
	Height-for-age z-score, mean (SD)^c^	−1.7 (2.3)	−1.7 (2.5)	0.030	−1.7 (1.8)	0.631
	Duration of breastfeeding in months, mean (SD)^c^	11.5 (6.2)	12.1 (6.2)	<0.001	11.4 (6.2)	0.377
	Received at least one dose PCV, n (%)	1021 (80.9%)	2268 (83.8%)	0.005	2062 (83.8%)	0.049
	Received at least two doses PCV, n (%)	886 (70.2%)	2030 (75.0%)	<0.001	1860 (75.6%)	<0.001
	Received three doses PCV, n (%)	611 (48.4%)	1414 (52.2%)	<0.001	1326 (53.9%)	<0.001

PCV indicates pneumococcal conjugate vaccine; IQR, interquartile range; SD, standard deviation; PCA, Principal Component Analysis.

a Cases versus clinic controls.

b Cases versus community controls.

c 326 clinic controls and 284 community controls missing maternal age; 2 clinic controls missing maternal education, 7 clinic controls and 5 community controls missing number of children <5 years old in household; 27 clinic controls and 31 community controls missing duration of breastfeeding in months, 9 clinic controls and 27 community controls missing height.

On the other hand, we found key differences between cases and community controls that we did not similarly observe between cases and clinic controls (Table 2). Cases, as compared to matched community controls, were 0.4 months younger. Unlike community controls, clinic controls lived on average 3.1 km closer to the clinic and breastfed for an average of 0.6 months longer than cases.

In our adjusted models based on all age-eligible cases and matched clinic controls (Table 3), we observed a vaccine effectiveness of 21.4% (95% confidence interval (CI 95%), −0.2%, 38.4%) for at least two PCV doses (primary analysis), and 23.5% (95% CI, −4.9%, 44.2%) for three PCV doses and 17.1% (95% CI, −3.8%, 33.8%) for at least one PCV dose (secondary analyses). When restricting the matched clinic control analysis to 3–11 month olds for secondary analyses, adjusted vaccine effectiveness estimates were 47.3% for three PCV doses (95% CI, 10.5%, 69.0%), 28.0% for at least two PCV doses (95% CI, −0.2%, 48.5%), and 21.3% for at least one PCV dose (95% CI, −5.8%, 41.5%). The 95% CI for the estimates of adjusted vaccine effectiveness among 12–35 month olds all crossed zero. Our adjusted tests for trend were statistically significant among all children and among 3–11 month olds using clinic controls, indicating increasing vaccine effectiveness with more PCV doses.

**Table 3:** PCV10 effectiveness against World Health Organization-defined radiographic pneumonia stratified by age and vaccine doses – Analysis based on clinic controls.

	Cases used in the analysis n (%)	Controls used in the analysis n (%)	Crude vaccine effectiveness (95% CI)	p value for trend – unadjusted	Cases used in the analysis n (%)	Controls used in the analysis n (%)	Adjusted vaccine effectiveness (95% CI)^a^	p value for trend – adjusted
All children				<0.001				0.004
Total	1262	2707			1262	2707		
Analysis – At least one dose	1245 (98.7%)	2707 (100%)	27.0% (9.3%, 41.2%)		1243 (98.5%)	2699 (99.7%)	17.1% (−3.8%, 33.8%)	
Analysis – At least two doses	1102 (87.3%)	2224 (82.2%)	29.6% (11.0%, 44.3%)		1100 (87.2%)	2218 (81.9%)	21.4% (−0.2%, 38.4%)	
Analysis – Three doses only	773 (61.3%)	1388 (51.3%)	33.4% (10.2%, 50.6%)		772 (61.2%)	1384 (51.1%)	23.5% (−4.9%, 44.2%)	
3–11 months				<0.001				0.007
Total	729	1583			729	1583		
Analysis – At least one dose	708 (97.1%)	1553 (98.1%)	37.2% (17.1%, 52.4%)		702 (96.3%)	1529 (96.6%)	21.3% (−5.8%, 41.5%)	
Analysis – At least two doses	584 (80.1%)	1158 (73.2%)	39.6% (17.6%, 55.7%)		579 (79.4%)	1137 (71.8%)	28.0% (−0.2%, 48.2%)	
Analysis – Three doses only	311 (42.7%)	503 (31.8%)	55.7% (28.3%, 72.6%)		310 (42.5%)	500 (31.6%)	47.3% (10.5%, 69.0%)	
12–35 months				0.252				0.607
Total	531	1110			531	1110		
Analysis – At least one dose	526 (99.1%)	1086 (97.8%)	4.59% (−35.3%, 32.5%)		526 (99.1%)	1084 (97.7%)	−2.1% (−46.6%, 28.8%)	
Analysis – At least two doses	505 (95.1%)	1013 (91.3%)	10.9% (−27.9%, 37.9%)		504 (94.9%)	1000 (90.1%)	3.2% (−40.9%, 33.6%)	
Analysis – Three doses only	449 (84.6%)	849 (76.5%)	8.0% (−35.3%, 37.5%)		449 (84.6%)	848 (76.4%)	0.1% (−50.1%, 33.2%)	

PCV indicates pneumococcal conjugate vaccine; CI, confidence interval.

a Adjusted for the following (10 variables): Father usually resides in the household, number of under-5 children in the household, mother or mother and someone else (not father) makes decisions about purchases for daily household needs, child goes into the cooking area while food is being cooked every day, house owned by household or household member, mother can take child when sick alone to the local welfare center or health complex, number of rooms in the house for household members to sleep in, traditional mud stove is used for cooking, father has greater than five years of education, continuous principal components analysis-derived socioeconomic measure of housing characteristics and assets.

When considering adjusted models with community controls (Table 4), the 95% CI in all models crossed zero, regardless of PCV dose number.

**Table 4:** PCV10 effectiveness against World Health Organization-defined radiographic pneumonia stratified by age and vaccine doses – Analysis based on community controls.

	Cases used in the analysis n (%)	Controls used in the analysis n (%)	Crude vaccine effectiveness (95% CI)	p value for trend – unadjusted	Cases used in the analysis n (%)	Controls used in the analysis n (%)	Adjusted vaccine effectiveness (95% CI)^a^	p value for trend – adjusted	
All children				<0.001%				0.232	
Total	1262	2461			1262	2461			
Analysis – At least one dose	1209 (95.8%)	2461 (100.0%)	19.7% (0.2%, 35.5%)		1204 (95.4%)	2443 (99.3%)	11.7% (−14.3%, 31.7%)		
Analysis – At least two doses	1064 (84.3%)	2048 (83.2%)	18.5% (−2.7%, 35.4%)		1059 (83.9%)	2032 (82.6%)	7.6% (−22.2%, 30.0%)		
Analysis – Three doses only	741 (58.7%)	1306 (53.1%)	2.2% (−31.6%, 27.3%)		741 (58.7%)	1303 (52.9%)	−15.1% (−65.4%, 19.9%)		
								
3–11 months				<0.001				0.204	
Total	729	1405			729	1405			
Analysis – At least one dose	691 (94.8%)	1394 (99.2%)	43.2% (24.2%, 57.5%)		686 (94.1%)	1379 (98.1%)	21.9% (−9.8%, 44.4%)		
Analysis – At least two doses	568 (77.9%)	1058 (75.3%)	43.9% (23.1%, 59.0%)		563 (77.2%)	1045 (74.4%)	17.7% (−20.1%, 43.6%)		
Analysis – Three doses only	296 (40.6%)	465 (33.1%)	36.3% (−4.5%, 61.2%)		296 (40.6%)	465 (33.1%)	−25.4% (−128%, 31.2%)		
12–35 months				0.836				0.879	
Total	531	1051			531	1051			
Analysis – At least one dose	511 (96.2%)	1007 (95.8%)	−25.6% (−77.4%, 11.1%)		510 (96.0%)	1004 (95. 5%)	−15.6% (−74.5%, 23.4%)		
Analysis – At least two doses	489 (92.1%)	935 (89.0%)	−25.6% (−79.9%, 12.3%)		488 (91.9%)	932 (88.7%)	−17.3% (−80.9%, 23.9%)		
Analysis – Three doses only	437 (82.3%)	805 (76.6%)	−22.0% (−78.5%, 16.7%)		436 (82.1%)	802 (76.3%)	−20.2% (−92.8%, 25 0.1%)		

PCV indicates pneumococcal conjugate vaccine; CI, confidence interval.

a Adjusted for the following (11 variables): Age at clinic visit, father usually resides in the household, number of under-5 children in the household, mother or mother and someone else (not father) makes decisions about purchases for daily household needs, child goes into the cooking area while food is being cooked every day, house owned by household or household member, mother can take child when sick alone to the local welfare center or health complex, number of rooms in the house for household members to sleep in, traditional mud stove is used for cooking, father has greater than five years of education, continuous principal components analysis-derived socioeconomic measure of housing characteristics and assets.

## 5 Discussion

This case-control study provides evidence for the effectiveness of PCV10, administered as a three dose primary series without a booster dose, in the prevention of WHO-defined radiographic pneumonia among children in rural Bangladesh during a routine immunization program. Our study analyzed vaccine effectiveness using clinic and community controls. We found vaccine effectiveness when using clinic controls but did not with community controls. Lack of vaccine effectiveness with community controls may have been driven by two main issues. First, our sample size calculation assumed an average of 46% vaccine coverage with two doses. We found that vaccine uptake was more rapid and less variable than anticipated with an average of 75% vaccine coverage, which reduced power and increased confidence interval widths. Second, information/measurement bias may have misclassified some children with pneumonia as controls during community control enrollment. Nevertheless, overall our study suggests that WHO Southeast Asian countries with similar *S. pneumoniae* epidemiology may expect vaccine effectiveness of PCV10 against radiographic pneumonia of about 21%, and possibly as high as 47% among 3–11 month old children who receive three PCV doses. These results are of additional importance given younger children are estimated to represent >80% of all child pneumonia deaths in LMICs [17], and the WHO Southeast Asian region contributes about 27% of the total global burden of pneumococcal mortality [4].

Our study’s estimated vaccine effectiveness of PCV against radiographic pneumonia using clinic controls, including the age patterns we observed, is consistent with other LMIC PCV efficacy trials and case-control studies that used a three dose primary series without a booster dose. In one of the only PCV trials from Asia to date, the authors studied the efficacy of a primary series of three 11-valent PCV doses against radiographic pneumonia among less than two year old children in the Philippines using a randomized placebo design [8]. Although the trial’s per protocol analysis found an overall vaccine efficacy of 22.9% (95% CI, −1.1%, 41.2%), the authors also reported higher PCV efficacy among 3–11 month olds (34.0% (95%, CI, 4.8%, 54.3%) than 12–23 month olds (2.7% (95% CI, −43.3%, 34.0%) [8]. A recent Gambian study used a case-control design with community controls [14]. While this study’s case control analysis was also under-powered in that it was designed to detect a minimum of 35% effectiveness, the authors reported PCV effectiveness estimates against radiographic pneumonia with a full three dose primary series of 28.0% (95% CI, −23.0%, 58.0%) [14]. However, this study’s vaccine effectiveness estimates also suggested better protection among younger children [14]. Three-dose vaccine effectiveness among 3–11 month olds was 43.0% (95% CI, −8.0%, 70.0%) and among ≥12 month olds was only 7.0% (95% CI, −264.0%, 76.0%) [14].

Although the potential benefit of a booster dose of PCV in protecting older children against radiographic pneumonia during real-world conditions is uncertain [18], current evidence shows that geometric mean antibody concentrations against vaccine serotypes are higher when dose three is administrated as a booster dose [19]. One recent case-control study from South Africa evaluated 13-valent PCV given as a two dose primary series with a booster dose at 10 months of age [15]. This study analyzed vaccine effectiveness using both hospital and community controls and reported results that may imply better protection against radiographic pneumonia among older children using this regimen [15].

Unlike clinic control analyses, we did not find vaccine effectiveness of PCV10 against radiographic pneumonia with community controls. Given the clinic controls analyses were consistent with other studies it is possible that these observed differences in the community control analyses were due in part to information or measurement bias that misclassified ineligible children, some of whom may have had radiographic pneumonia, as community controls. It is difficult to accurately quantify how much this may have biased the community control analyses. Multiple factors support the presence of some degree of information or measurement bias within the community control data. First, children eligible to be clinic controls were evaluated by study physicians while children eligible to be community controls were evaluated by CHWs. Although the screening processes administered by study physicians and CHWs were similar, CHWs were not formal healthcare providers. Despite intensive efforts to standardize CHWs clinical assessments, their examinations were likely less accurate than study physicians, and some community controls may have had pneumonia. Quality assurance assessments of CHW performance during the study period suggest this was a possible issue. We found that among 62 children assessed by study physicians to have pneumonia, CHWs classified 56.4% (n = 35) of the same children as not having pneumonia. Second, study physicians were masked to the child’s case-control status since at the time of their evaluation, the child had not yet had a chest radiograph. CHWs, on the other hand, enrolled potential controls and were therefore not masked to case-control status of the child and this may have biased their assessment. Third, it is also possible that some community controls may have been previously ill with pneumonia but this was unrecognized since, unlike clinic controls, they may have sought care outside of study clinics. Data collected prior to the study suggests that the community control analysis was indeed vulnerable to care seeking outside of study clinics. Between January 2014 and June 2015 we found in this study population only 12.4% (1216/9850) of children below age five years with suspected pneumonia sought care at study clinics. Non-study clinics may have in turn prescribed antibiotics and not informed the caregiver that the treatment was for pneumonia or, alternatively, they may not have recognized the child to have pneumonia but nevertheless prescribed antibiotics that treated it. These issues may have been compounded by the fact that CHWs used the caregiver’s one week recall to assess the child for community control eligibility, but eligibility assessments were on average completed nearly two weeks after cases. In contrast, clinic control assessments did not rely on caregiver recall since study physicians excluded pneumonia by examining the child. The potential effect of this type of differential misclassification may have been an important explanation for the results we obtained with our analysis of community controls.

Evaluating vaccine effectiveness using a case-control design can also inherently underestimate the vaccine’s protective effect due to unmeasured confounders. Given our clinic control results are consistent with vaccine effectiveness estimates from other PCV studies among different populations, we think any bias from residual confounding is likely to be minimal in the clinic control analyses. However, residual confounding or unmeasured heterogeneity may be another reason for this study’s lack of vaccine effectiveness when using community controls. Children who sought care at the Upazila Health Complex outpatient clinics may be inherently different from those who did not and our dataset may not have captured those differences.

One prior case-control study from Bangladesh that evaluated the vaccine effectiveness of the *Haemophilus influenzae* type b (Hib) vaccine against radiographic pneumonia among children found similarly discordant results using a comparable hospital and community control identification approach [20]. In this study the authors reported an adjusted vaccine effectiveness following at least two Hib doses of 17.0% (95% CI, −10.0%, 38.0%) using community controls and 35.0% (95% CI, 13.0%, 52.0%) with hospital controls [20]. Taken together with our findings, this may suggest that hospital or clinic controls in pluralistic South Asian healthcare systems with complex care seeking patterns may not only be more representative of all controls in the study population but they can also be more reliably studied.

Deploying formal health care workers in the field to evaluate community controls, or extensive training of CHWs, could lower information and classification bias. We recommend other studies in similarly complex settings consider focusing on hospital and/or clinic controls given our clinic control results were consistent with the literature and we faced challenges enrolling community controls.

This study has another notable limitation. WHO-defined radiographic primary endpoint pneumonia is a non-specific endpoint for vaccine effectiveness against *S. pneumoniae* and interpretations of chest radiography images are known to have inter- and intra-observer variation [21], [22]. However, the WHO radiographic pneumonia definition has been used for nearly two decades for vaccine efficacy studies of bacterial conjugate vaccines and child pneumonia epidemiological research, and our use of this endpoint increases the generalizability of our findings. In addition, our application of the WHO methodology was done rigorously and under close quality control. We have previously reported elsewhere the detailed performance of our study reading panel and quality control measures [23]. In brief, the primary chest radiograph readers in our study agreed on the presence or absence of WHO-defined radiographic primary endpoint pneumonia in 79% of 9533 interpretable images. Quality control expert readings agreed with overall reading panel classifications (presence/absence of WHO-defined radiographic pneumonia) on 92.9% of a random subset of 1652 images. This overall performance is consistent with other contemporary child pneumonia studies that have utilized reading panels to interpret chest radiographs using the WHO methodology [22].

In conclusion, despite challenges enrolling and analyzing community controls our clinic control results support PCV introduction throughout Bangladesh and Southeast Asia while also informing future study designs in complex, pluralistic healthcare settings. National policy makers and program managers in Bangladesh and other neighboring countries should consider these findings as they graduate from Gavi funding and decide how to best allocate their healthcare resources to benefit the health of children.

## Declaration of Competing Interest

The authors declare the following financial interests/personal relationships which may be considered as potential competing interests: [EDM, SA, NHC, SJR, AMK, ADR, AAMH, FP, NS, MER, MH, HBS, AQ, NB, MS, LHM, WC, and AHB reports grants from Bill and Melinda Gates Foundation, grants from GlaxoSmithKline, during the conduct of the study.].
